# Pharmacokinetic-Pharmacodynamic Relationship of Erenumab (AMG 334) and Capsaicin-Induced Dermal Blood Flow in Healthy and Migraine Subjects

**DOI:** 10.1007/s11095-017-2183-6

**Published:** 2017-06-07

**Authors:** Thuy Vu, Peiming Ma, Jiyun Sunny Chen, Jan de Hoon, Anne Van Hecken, Lucy Yan, Liviawati Sutjandra Wu, Lisa Hamilton, Gabriel Vargas

**Affiliations:** 1 Clinical Pharmacology, Modeling and Simulation, Amgen Inc., One Amgen Center Drive, Thousand Oaks, California, 91320-1799 USA; 2 Clinical Pharmacology, GSK R&D, Shanghai, China; 3 Medivation, San Francisco, California, USA; 4 Center for Clinical Pharmacology, University Hospitals of Leuven, Leuven, Belgium; 5 Global Biostatistical Sciences, Amgen Limited, Uxbridge, England UK

**Keywords:** anti-CGRP receptor, dermal blood flow, migraine, pharmacokinetics-pharmacodynamics, vasodilation

## Abstract

**Purpose:**

Capsaicin-induced dermal blood flow (CIDBF) is a validated biomarker used to evaluate the target engagement of potential calcitonin gene-related peptide-blocking therapeutics for migraine. To characterize the pharmacokinetics (PK) and quantify the inhibitory effects of erenumab (AMG 334) on CIDBF, CIDBF data were pooled from a single- and a multiple-dose study in healthy and migraine subjects.

**Methods:**

Repeated capsaicin challenges and DBF measurements were performed and serum erenumab concentrations determined. A population analysis was conducted using a nonlinear mixed-effects modeling approach. Effects of body weight, gender, and age on model parameters were evaluated.

**Results:**

Two-compartment target-mediated drug disposition (TMDD) model assuming binding of erenumab in the central compartment best described the nonlinear PK of erenumab. Subcutaneous absorption half-life was 1.6 days and bioavailability was 74%. Erenumab produced a maximum inhibition of 89% (95% confidence interval: 87–91%). Erenumab concentrations required for 50% and 99% of maximum inhibition were 255 ng/mL and 1134 ng/mL, respectively. Increased body weight was associated with increased erenumab clearance but had no effect on the inhibitory effect on CIDBF.

**Conclusions:**

Our results show that erenumab pharmacokinetics was best characterized by a TMDD model and resulted in potent inhibition of CIDBF.

**Electronic supplementary material:**

The online version of this article (doi:10.1007/s11095-017-2183-6) contains supplementary material, which is available to authorized users.

## Introduction

Migraines are episodic headaches that are sometimes preceded by sensory warning symptoms or signs (auras) and are often accompanied by nausea, vomiting, and extreme sensitivity to light (photophobia) and sound (phonophobia). There is a great unmet medical need for effective migraine therapies, especially for prophylactic treatment. Over 36 million Americans (12% of the population of the United States) suffer from migraine attacks, with approximately 4 million patients enduring severe impairment and requiring bed rest for 4 or more days per month. Results from recent large-scale headache questionnaires indicate that approximately 14 million migraineurs in the United States would qualify for and benefit from an effective and safe preventive therapy [1–3]. Globally, it is believed that migraine affects more than 10% of the world’s population and causes substantially more individual morbidity [4]. The debilitating effects of migraine have a substantial economic impact on the individual and society, and cause significant social and school−/work-related losses.

Calcitonin gene-related peptide (CGRP) is a neuropeptide and vasodilator, which has been implicated as a key mediator in the initiation and progression of migraine pain. Results from clinical studies with small molecule CGRP-antagonists (e.g., olcegepant and telcagepant) as well as monoclonal antibodies (mAbs) neutralizing CGRP or blocking its receptor, have demonstrated that targeting the CGRP pathway provides clinical benefit to individuals with migraine. Given the efficacy of CGRP-blocking therapeutics both in the treatment of acute migraine pain and in prevention, the so-called capsaicin model was developed to assess target engagement for the evaluation of these compounds in early clinical development. The capsaicin model involves the topical application of capsaicin on the human forearm skin. By activating the transient receptor potential vanilloid type 1 (TRPV1) receptor on nociceptive nerve endings, capsaicin induces the local release of CGRP and increases the dermal blood flow (DBF), which can be measured using laser Doppler imaging. The capsaicin-induced DBF (CIDBF) model is well validated, non-invasive, and technically uncomplicated [5]. As it has previously been shown to be a robust model and allows repeated measurements which are reproducible over time, it facilitates the early clinical evaluation of CGRP-blocking therapeutics [6].

Erenumab (AMG 334) [7] is a mAb that binds to the CGRP receptor and thus functions as a competitive inhibitor of the native ligand CGRP. In vitro data have shown that erenumab binds to human CGRP receptors with high affinity (dissociation equilibrium constant K_D_ = 20 pM) and potency (IC_50_ = 2.3 nM) in a competitive and reversible manner [8]. Results from single-ascending dose (SAD) and multiple-ascending dose (MAD) phase 1 studies (NCT01688739 and NCT01723514, respectively) have been reported and showed that erenumab inhibited CIDBF in healthy and migraine subjects, indicating CGRP-receptor antagonism [9] (de Hoon JN, Van Hecken A, Yan L, Smith B, Chen JS, Bautista E, et al., unpublished data). Results from non-compartmental analysis suggested nonlinear pharmacokinetic (PK) characteristics for erenumab; namely, a decreased clearance with increased dose, which is typical of target-mediated drug disposition (TMDD) for mAb therapeutics [10, 11].

This article reports the results of a population PK-pharmacodynamics (PD) analysis that quantified the relationship between serum erenumab concentrations and inhibition of CIDBF in healthy subjects and migraine patients. The objectives of this analysis were to: 1) characterize the time course of serum erenumab concentrations following subcutaneous (SC) and intravenous (IV) administrations using a TMDD model; 2) quantify the inhibitory effect of systemic exposure of erenumab on CIDBF; and 3) evaluate the effect of baseline characteristics and other variables as potential sources of variability in PK and PD parameters for erenumab. The modeling results would be used to design rational dose regimens for a phase 2 study evaluating safety and efficacy of erenumab in the target population of patients with migraine.

## Materials and Methods

### Study Design

PK and DBF data were pooled from a SAD study and a MAD study for population PK-PD analysis. Inclusion/exclusion criteria and assessments for these two clinical studies have been reported [12, 13]. The SAD study was a double-blind, placebo-controlled, sequential dose escalation study, in which 60 subjects were enrolled. Forty-eight healthy subjects were randomized in a 3:1 ratio (erenumab:placebo) to receive erenumab or placebo, where the erenumab treatment was either SC administration of 1, 7, 21, 70, 140, or 210 mg or IV of 140 mg. In addition, a total of 12 migraine subjects were randomized in a 1:1 ratio to receive a SC dose of 140 mg erenumab or placebo. The MAD study was a double-blind, placebo-controlled study in which 48 subjects were enrolled. Thirty-two healthy subjects were randomized in a 3:1 ratio (erenumab:placebo) to receive erenumab SC 21, 70, 140, 210 mg x 3 doses, 280 x 1 dose + 210 mg x 2 doses, or 3 doses of placebo; 16 migraine subjects were randomized in a 3:1 ratio (erenumab:placebo) to receive erenumab SC 21 or 140 mg x 3 doses. Each erenumab or placebo dose was administered every 4 weeks (Q4W). Sampling schedules for collections of PK and DBF measurements are shown in Table I. In both the SAD and MAD studies, erenumab was administered to all subjects (healthy subjects and migraine patients) at protocol-specified visits regardless of the timing of migraine attacks [12, 13].

**Table I:** Summary of Pharmacokinetic and Pharmacodynamic Studies

Study code	Dose cohorts (mg)	Number of subjects	PK sampling times	PD sampling times
Single ascending dose		60		
Healthy subjects	Placebo, 1, 7, 21, 70, 140, or 210 mg SC; 140 mg IV	48	Days 1 (pre-dose and 0.5^a^, 1^a^, and 8 h post-dose), 2–5, 8, 12, 15, 22, 29, 43, 57, 64, 85, 99, and 127	At screening (days −21 to −2^b^, and day −1) and days 2^c^, 4^c^, 15, 29, 43, 64, 85, 99, 127, and 155
Migraine patients	Placebo or 140 mg SC	12		
Multiple ascending dose		48		
Healthy subjects	Placebo, 21, 70, 140 mg SC;	32	Days 1 (pre-dose and 8 h post-dose), 4, 5, 8, 12, 15, 22, 29 (pre-dose), 36, 57^d^ (pre-dose and 8 h post-dose), 64, 71, 85, 99, 113, 127, 169^d^, 197^d^, and 224	At screening (days −35 to −2) and days −1, 8, 57 (pre-dose), 85, 113, 169, and 197
	280 mg x 1 + 210 mg x 2 SC			
	Q4W x 3 doses			
Migraine patients	Placebo, 21, or 140 mg SC	16		
	Q4W x 3 doses			

^a^PK sample collection time points required for IV cohort only

^b^For IV cohort, screening DBF performed between days −21 to −4

^c^DBF performed on day 2 for IV cohort and on day 4 for SC cohort

^d^PK sample collection coincided with dermal blood flow measurement

*DBF* Dermal blood flow, *IV* Intravenous, *PD* Pharmacodynamic, *PK* Pharmacokinetic, *Q4W* Every 4 weeks, *SC* Subcutaneous

Studies were conducted at a single center in Leuven, Belgium, approved by the independent ethics committee affiliated with the study center, and performed in accordance with the Declaration of Helsinki. All subjects provided written informed consent.

### Bioanalytical Method

Erenumab in human serum was quantified according to the validated analytical procedure for the quantification of erenumab in human serum that was developed at Amgen Inc. (Thousand Oaks, CA, USA). Standards (STDs) and quality controls (QCs) were prepared by spiking erenumab into 100% human serum. STD, QC, blank, and study samples were added to a plate that had been passively coated with a mouse anti–erenumab monoclonal antibody (mAb). After capture of erenumab to the immobilized antibody, unbound materials were removed by a wash step. Biotin-conjugated mouse anti–erenumab mAb was added for detection of captured erenumab. After another incubation step with streptavidin-HRP, a tetramethylbenzidine peroxide substrate solution was added to produce a colorimetric signal that was proportional to the amount of erenumab bound by the capture reagent. The color development was stopped by addition of sulfuric acid, and the instrument response was measured at 450 nm with reference to 650 nm. The instrument response vs concentration relationship was regressed according to a four-parameter logistic (Marquardt) regression model with a weighting factor of 1/Y^2^. The conversion of instrument response for QCs and study samples to concentrations was performed using Watson LIMS (v7.4; Thermo, PA, USA) data reduction software. The lower and upper limits of quantification were 1.00 ng/mL and 100.00 ng/mL, respectively.

### Dermal Blood Flow Measurements

In both clinical studies, the CIDBF PD assay as described by Van der Schueren et al. [5] was used to determine CGRP receptor antagonism. Briefly, subjects received topical doses of 1000 μg capsaicin per 20 μL vehicle (i.e., a 3:3:4 mixture of ethanol 100%, Tween-20, and distilled water). Capsaicin was applied at two sites on the volar surface of a subject’s left or right forearm, and vehicle only was applied to one site on the volar surface of the same arm as a control. DBF was assessed by laser Doppler perfusion imaging (Periscan PIM III, Perimed AB, Sweden) and was done immediately before (i.e., “baseline” perfusion) and 0.5 h after capsaicin application on the skin at these three sites. DBF measurements were performed before erenumab dosing (i.e., pre-dose) and at pre-specified time points after dosing as outlined in Table I.

### PK and PD Model Development

#### PK Model

The schematic for the PK-PD model is shown in Supplementary Fig. S1. A two-compartment TMDD model was used to describe the PK of erenumab after SC and IV administrations. SC absorption of erenumab was described with first-order absorption rate. Unbound erenumab concentrations were eliminated from the central compartment via a linear elimination pathway and a saturable elimination pathway attributed to the target-mediated mechanism that was described by a Qss model [14]. The equations used to describe the PK system were as follows:

1 $$ \begin{array}{l}\frac{d{A}_{SC}}{ d t}=-{k}_a\cdot \kern0.5em {A}_{SC}\\ {}\kern0.5em {A}_{SC}(0)=\frac{1}{1+ \exp (F)}\cdot \kern0.5em {Dose}_{SC}\end{array} $$

where F estimate is from negative infinity to positive infinity and the ratio 1/(1 + exp(F)) is the total fraction of bioavailable dose; k_a_ is the first-order absorption rate constant; A_SC_ is the amount absorbed; and Dose_SC_ is the SC dose administered at time *t* = 0. The Qss constant (K_ss_) was estimated directly from the available unbound serum erenumab concentration data, which is derived as:

2 $$ {\left[ AMG334\right]}_{\mathrm{unbound}}=0.5\cdot \left[\left(\frac{{\mathrm{A}}_{tot}}{V_c}-{R}_{tot}(t)-{K}_{ss}\right)+\sqrt{{\left(\frac{A_{tot}}{V_c}-{R}_{tot}(t)-{K}_{ss}\right)}^2+4\cdot {K}_{ss}\cdot \frac{A_{tot}}{V_c}}\right] $$

Where R_tot_(t) is determined using differential equation $\frac{d{ R}_{tot}}{ d t}={k}_{syn}-{k}_{\deg}\bullet {R}_{tot}-\left({k}_{int}-{k}_{\deg}\right)\bullet \left(\frac{A_{tot}}{V_c}-{\left[\mathrm{AMG}334\right]}_{\mathrm{unbound}}\right)$, with initial condition R_tot_(0) = k_syn_/k_deg_, k_syn_ is the zero-order receptor production rate and k_deg_ is the first-order receptor degradation rate. The total erenumab amount in the central compartment (A_tot_) and unbound erenumab amount in the peripheral compartment (A_p_) are estimated using differential equations as follows:

3 $$ \frac{dA_{tot}}{dt}={k}_a\bullet {A}_{SC}+{k}_{p c}\bullet {A}_p-{k}_{int}\bullet {A}_{tot}-\left({k}_{c p}+{k}_{c0}-{k}_{int}\right)\bullet {\left[\mathrm{AMG}334\right]}_{\mathrm{unbound}}\bullet {V}_c $$

4 $$ \frac{d{ A}_p}{ d t}={k}_{c p}\bullet {\left[\mathrm{AMG}334\right]}_{\mathrm{unbound}}\bullet {V}_c-{k}_{p c}\bullet {A}_p $$

where the initial conditions are A_tot_(0) = Dose_IV_ and A_p_(0) = 0; V_c_ is the volume of distribution in the central compartment; k_int_ is the internalization rate constant for bound erenumab; and k_pc_, k_cp_, and k_c0_ are the peripheral to central compartment, central to peripheral compartment, and linear elimination rate constants, respectively.

The total systemic clearance for erenumab was computed with Eq. 5, where CL_linear_ is a parameter estimated by the PK model and CL_nonlinear_ is a parameter derived from a combination of target-mediated parameters (R_tot,ss_, K_ss_, and k_int_) estimated by the PK model. CL_nonlinear_ is derived using Eq. 6 under the Qss assumption [14, 15].

5 $$ {CL}_{total}={CL}_{linear}+{CL}_{nonlinear} $$

6 $$ {CL}_{nonlinear}=\frac{k_{int}}{K_{ss}}\cdot {V}_c\cdot {R}_{tot, ss}\cdot \left(1-\frac{AMG334}{AMG334+{K}_{ss}}\right) $$

#### PD Model

Individual PK parameters from the PK model were used to generate concentrations as predictor of CIDBF. The response variable was ΔDBF_t_, which was computed as the difference of DBF measurements after and before capsaicin challenge at time t (e.g., day after first dose). After correcting for baseline ΔDBF on day 0, the erenumab effect on DBF after capsaicin challenge was estimated. Serum concentration of erenumab in the central compartment was either indirectly or directly linked to the DBF changes over time, proportional to baseline DBF on day 0 (ΔDBF_0_) as follows:

7 $$ {\varDelta DBF}_t={\varDelta DBF}_0\bullet \left(1-\frac{I_{\max}\bullet {\left[\mathrm{AMG}334\right]}^{hill}}{{{I C}_{50}}^{hill}+{\left[\mathrm{AMG}334\right]}^{hill}}\right) $$

where I_max_ is the maximum inhibition of DBF due to erenumab treatment, IC_50_ is a potency parameter or estimate of serum erenumab concentration at 50% of I_max_ and erenumab is the serum concentration in the central compartment. To assess the threshold (or on/off) effect of erenumab on DBF, a sigmoidicity parameter or hill coefficient was also estimated. To estimate the erenumab concentration needed to achieve maximum possible DBF inhibition, IC_99_ was derived as 99^1/hill^·IC_50_.

The observed erenumab inhibitory effect on capsaicin-induced change in DBF was reported as the percent change in ΔDBF_t_ relative to ΔDBF_0_, i.e., inhibition (%) = 100·(ΔDBF_t_/ΔDBF_0_–1). The model predicted erenumab inhibition was defined by the I_max_·[erenumab]^hill^/(IC_50_^hill^ + [erenumab]^hill^) term in Eq. 7 above.

Details of additional methods including statistical model, covariate analyses, model evaluation criteria, simulations, and software used are provided as supplemental material (see Supplementary Methods).

## Results

### Subjects and Data

The pooled analysis dataset had PK-PD data for 108 subjects: 60 in the SAD study and 48 in the MAD study. Erenumab concentration data were available for 78 subjects and repeated-measurements of DBF data were available for 100 subjects, 30 of whom received placebo.

Of the 108 subjects, 80 (74%) were healthy subjects and 28 (26%) were migraine patients; 78% were male. The mean (standard deviation, SD) age of all subjects was 29.5 (9.7) years and the mean (SD) weight was 74.9 (11.9) kg (Table II).

**Table II:** Characteristics of Treated Subjects Included in the Pharmacokinetic-Pharmacodynamic Analysis

Parameter	Single-dose study	Multiple-dose study	All
n (%)	*N* = 60	*N* = 48	*N* = 108
Sex			
Male	51 (85)	33 (69)	84 (78)
Female	9 (15)	15 (31)	24 (22)
Study subjects			
Healthy subjects	48 (80)	32 (67)	80 (74)
Migraine patients	12 (20)	16 (33)	28 (26)
SC dose groups			
Erenumab	36 (60)	36 (75)	72 (67)
Placebo	16 (27)	12 (25)	28 (26)
IV dose groups			
Erenumab	6 (10)	–	6 (5)
Placebo	2 (3)	–	2 (2)
Mean ± SD			
Age (years)	27.2 ± 7.3	32.4 ± 11.4	29.5 ± 9.7
Male	27.0 ± 6.8	31.4 ± 10.1	–
Female	28.1 ± 10.4	34.5 ± 14.0	–
Body weight, (kg)	76.7 ± 11.1	72.7 ± 12.7	74.9 ± 11.9
Male	79.2 ± 9.3	76.1 ± 12.4	–
Female	62.2 ± 9.8	65.3 ± 10.1	–

N = number of subjects evaluated in each study; n = number of subjects for each parameter

*IV* Intravenous, *SC* Subcutaneous, *SD* Standard deviation

A total of 1297 erenumab serum concentrations from 78 subjects were available for population PK analysis, and 676 DBF measurements from 100 subjects were available for population PK-PD analysis. Of these samples, 82 (6%) PK measurements below the limit of quantification were excluded from the analysis. A total of 6 subjects developed binding anti-erenumab antibodies during the studies (1 in SAD and 5 in MAD); 1 subject (in the MAD study) was positive for neutralizing antibodies. The effect of anti-erenumab binding antibodies on PK parameters was not assessed due to a small number of observations.

### Pharmacokinetic Model

The population parameter estimates from a final two-compartment TMDD model with first-order absorption for SC administration is shown in Table III. To avoid model over-parameterization, the quasi-steady-state (Qss) model under the Qss assumption was used as an approximation of the general two-compartment TMDD model [14, 16]. The target receptor concentration was estimated to change over time.

**Table III:** Pharmacokinetic Parameter Estimates

Parameters	Units	Mean estimate	Bootstrapped estimate (95% CI)	Shrinkage (%)
Nonspecific linear clearance (CL)^a^	L/day	0.214	0.213 (0.191, 0.243)	
Central volume of distribution (V_c_)^a^	L	4.27	4.2 (3.4, 5.38)	
Intercompartmental clearance (Q)	L/day	3.34	3.25 (1.41, 6.79)	
Peripheral volume (V_p_)	L	2.73	2.75 (2.2, 3.18)	
Absorption rate (k_a_)	1/day	0.432	0.428 (0.346, 0.519)	
Bioavailability (F)	%	74	74 (66, 85)	
Receptor production rate (k_syn_)	ng/mL/day	51.9	53 (40.6, 65.4)	
Receptor degradation rate (k_deg_)	1/day	0.22	0.222 (0.144, 0.427)	
Binding affinity (K_ss_)	ng/mL	18.8	18.1 (10.6, 30.6)	
Internalization rate (k_int_)	1/day	0.0345	0.0347 (0.0251, 0.0442)	
BSV in CL	%CV	25.1	25 (21, 29.6)	4.3
BSV in V_c_	%CV	43.8	43.8 (35.3, 53)	5.6
BSV in k_a_	%CV	59.5	58.8 (45.6, 73.3)	7.9
BSV in k_int_	%CV	42.3	42.1 (33.5, 51.7)	6.8
Covariance between ηCL and ηk_a_	-	−0.0731	−0.0719 (−0.127, –0.0371)	
Covariance between ηCL and ηk_int_	-	0.0682	0.0664 (0.0396, 0.101)	
Residual variability	%CV	17.9	17.6 (15.9, 19.7)	10.7

95% CI = 95% confidence interval from 1000 bootstrapped runs of the final model

^a^Mean CL and V_c_ estimates at 70 kg; individual body weight effect on CL and V_c_ were estimated as Individual CL = 0.214 (weight/70)^0.75^ L/day and Individual V_c_ = 4.27 (weight/70) L

*BSV* Between-subject variability expressed as %CV, *CV* Approximates coefficient of variation

The linear non-specific clearance was estimated to be 0.214 L/day (between subject variability [BSV]: 25%) and the central volume of distribution was 4.27 L (BSV: 44%). The rate constant of the complex internalization (k_int_: 0.0345 day^−1^) was similar to the linear elimination of free erenumab (CL/V_c_: 0.0501 day^−1^). Maximal nonlinear clearance, derived from k_int_∙V_c_∙R_tot,ss_/K_ss_, was approximately 1.84 L/day and 8 times the linear clearance (Supplementary Fig. S2).

The precision of fixed- and random-effect parameter estimates was acceptable. The largest BSV estimate was in the absorption rate k_a_ (60%). Residual variability in erenumab concentrations was low—approximately 18% of the predicted concentrations, with a reasonable shrinkage of 11%. Correlations were observed in the random effects for PK parameters, −0.49 between k_a_ and CL, and 0.642 between CL and k_int_.

**Fig. 1:**
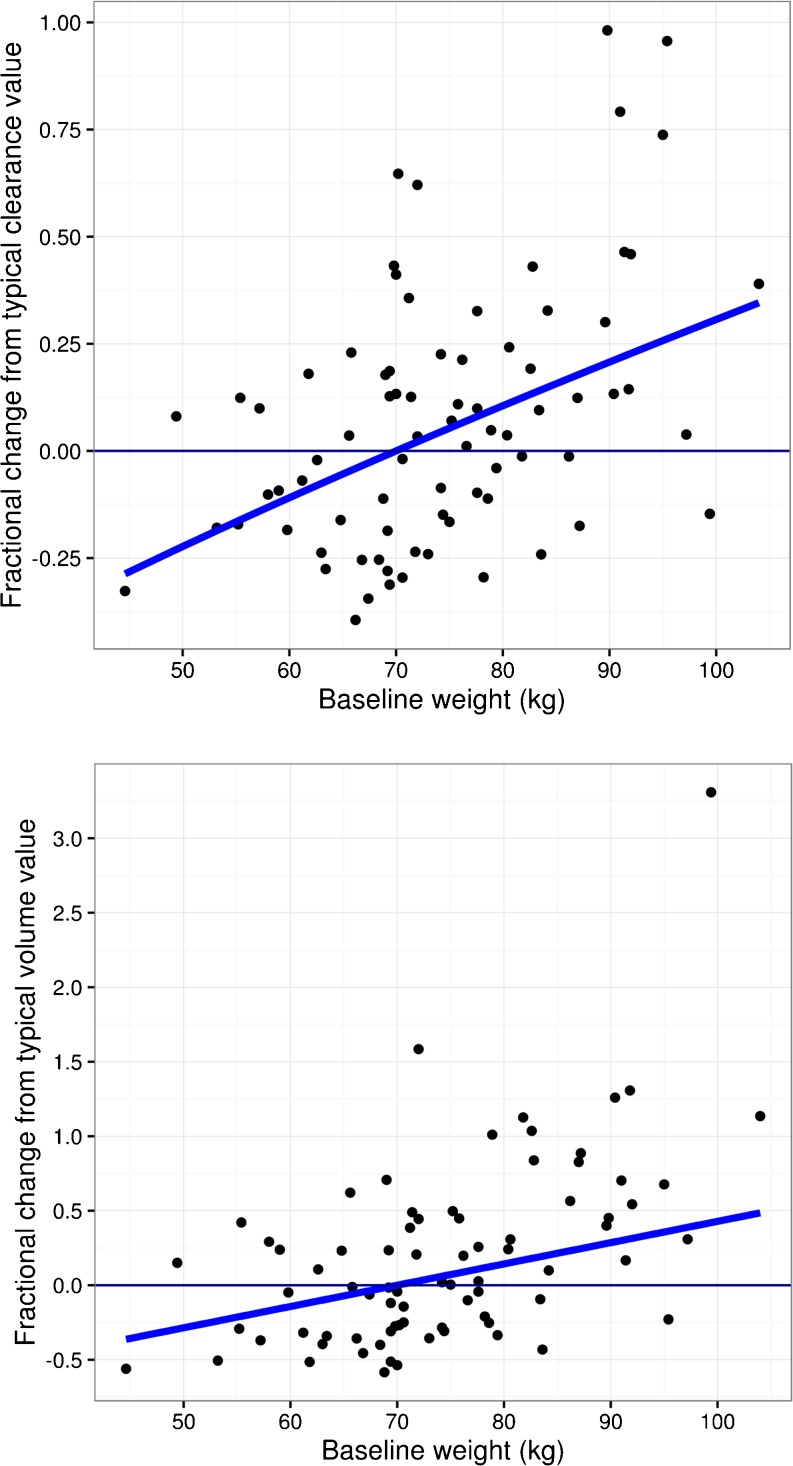
Relationship between body weight and pharmacokinetic parameters. Solid lines are typical population trends between parameter estimates and body weight; solid symbols are individual parameter estimates as function of body weight.

**Fig. 2:**
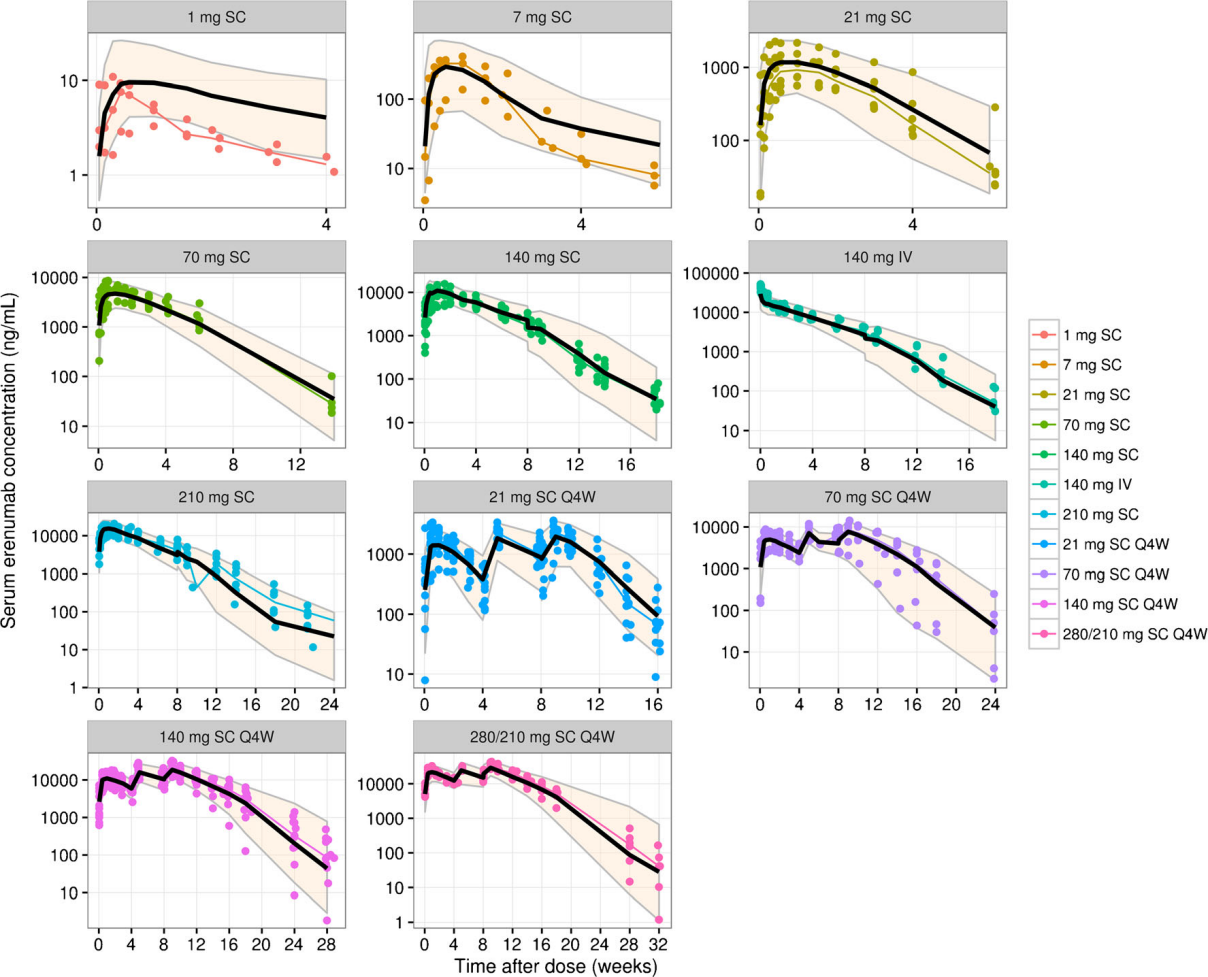
Visual predictive checks for the final pharmacokinetic model of erenumab concentrations. Colored symbols and lines are individual observations and the medians of the observations for the corresponding dosing cohorts; solid black lines and shaded bands are the model predicted 50th and 90th percentiles. IV, intravenous; Q4W, every 4 weeks; SC, subcutaneous.

Population predictions were significantly improved for the best-fitted model with CL and V_c_ adjusted for body weight (*p* < 0.001). The exponents of the effect of body weight on CL and V_c_ were fixed to allometric exponents of 0.75 and 1, respectively. Model fitting was not significantly better when estimating the exponents than when fixing them. When estimated, the exponents were 0.532 and 1.64 for CL and V_c_, respectively, and the change in minimum value of objective function was not statistically significant when comparing to the model with fixed allometric exponents (*p* > 0.001). Since all migraine patients were female, evaluation of sex covariate was the same as that of population (e.g., migraine vs. healthy). Sex and age, after adjusting for body weight, did not show statistical significances at *p* < 0.001 on CL or V_c_ as covariates when estimated. Consistent with observed data, erenumab serum concentration time courses were similar between healthy subjects and migraine patients (Supplementary Fig. S3). Fig. 1 shows the approximately linear relationships between body weight and the individual PK parameters, linear clearance and central volume of distribution. Goodness-of-fit plots (observed vs population and individual predicted concentrations, and residual vs time) did not show systematic bias in model predictions (Supplementary Figs. S4 and S5). The visual predictive check plots as an internal model evaluation showed that the distributions of observed and predicted concentrations were mostly in agreement (Fig. 2), except for lower doses (1 and 7 mg) because of the small sample size (*N* = 3 for each dose); uncertainty in predictions for the nonlinear concentration range (< R_tot_) was seen at higher doses, which reflects the lack of PK information by study design. Nevertheless, prediction corrected visual predictive check (VPC) clearly supported the appropriateness of the PK model after removing the variability largely due to limited data at lower doses/concentrations (Supplementary Fig. S6). Overall, the model captured population PK characteristics and variability across a wide range of doses and in both healthy and migraine populations.

### Pharmacodynamic Models

**Fig. 3:**
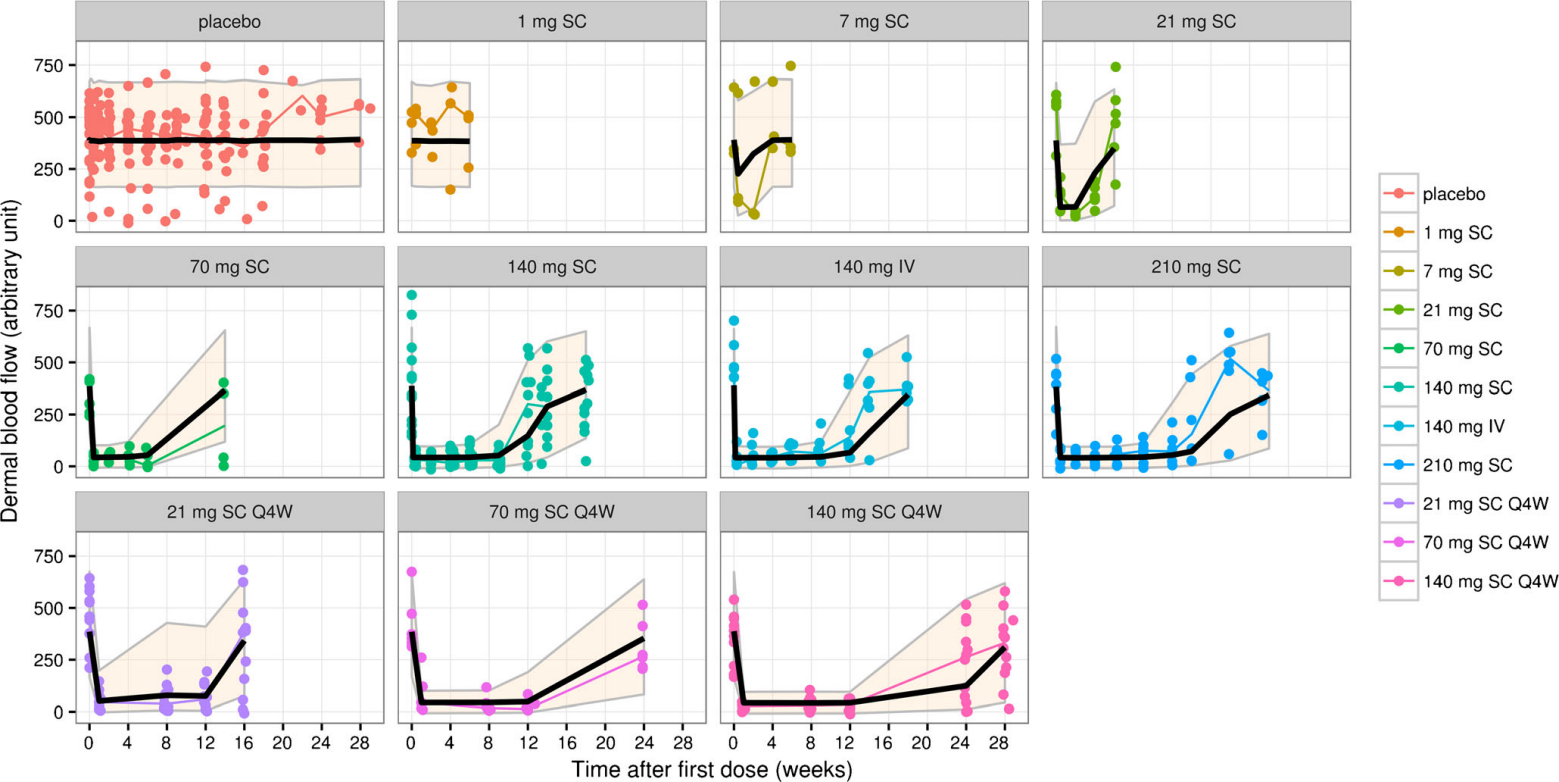
Visual predictive checks for the final pharmacodynamic model of dermal blood flow for erenumab. Colored symbols and lines are individual observations and the medians of the observations for the corresponding dosing cohorts; solid black lines and shaded bands are the model predicted 50th and 90th percentiles. IV, intravenous; Q4W, every 4 weeks; SC, subcutaneous.

**Fig. 4:**
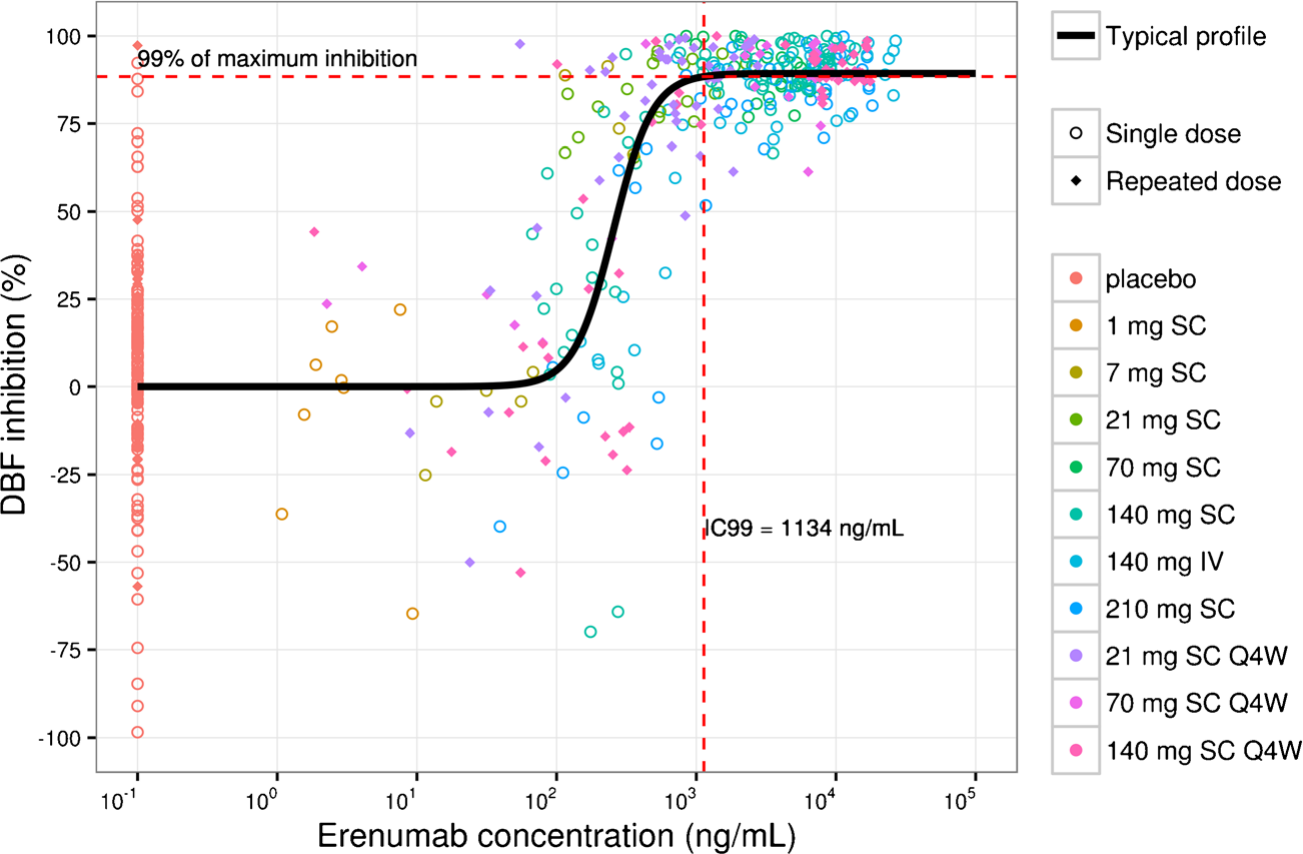
Relationship between erenumab concentrations and dermal blood flow (DBF) inhibition. The symbols represent individual observations. IV, intravenous; Q4W, every 4 weeks; SC subcutaneous.

A simple inhibitory E_max_ model was used to describe the effect of erenumab on the time course of DBF. Erenumab is assumed to have no effect on DBF over time in the absence of capsaicin challenge (e.g., at 0 min) but decreases DBF after capsaicin challenge (e.g., at 30 min). This is consistent with erenumab’s mechanism of action; it attenuates the action of CGRP in the periphery. The time courses of DBF inhibition were similar between healthy subjects and migraine patients (Supplementary Fig. S3). The assumption was verified by the observed data as shown in Supplementary Fig. S7, where DBF before capsaicin challenge did not change as erenumab concentration increased over time. The PD model adequately described the observed DBF time course in both single- and multiple-dose studies (Fig. 3). Prediction-corrected VPC further supported the appropriateness of the PD model to describe DBF time courses across a wide range of doses in phase 1 studies (Supplementary Fig. S6). After adjusting for body weight effect on PK, body weight had no additional effect on PD parameters. Deterministic simulations showed that the DBF inhibition is at maximum for the entire dosing interval at steady-state for the low (25th percentile) and high (75th percentile) body weights, after repeated dosing of 21 mg and 70 mg SC Q4W (Supplementary Fig. S8). All final PD parameter estimates were estimated with good precision (Table IV). Capsaicin challenge increased DBF on average by 398 (95% CI: 369–428) perfusion units (PU) with a BSV of 28% CV in the absence of erenumab; i.e., on average, DBF post-capsaicin challenge (at 30 min) increased approximately 10- to 12-fold above DBF pre-capsaicin challenge (at 0 min) in the absence of erenumab. The concentration-response curve was sigmoidal and plateaued at approximately 1134 ng/mL (IC_99_; Fig. 4). Change in DBF response is highly variable across concentration ranges and BSV in IC_50_ is large (%CV: 94, 95% CI: 68–120%). The hill coefficient was estimated to be 3.08 (95% CI: 0.555–5.61). Maximum inhibition of DBF corrected for baseline was approximately 89% with good precision (95% CI: 87–91%).

**Table IV:** Pharmacodynamic Parameter Estimates

Parameter	Units	Mean estimate (95% CI)	Shrinkage (%)
Baseline dermal blood flow	PU	398 (369, 428)	
Potency (IC_50_)	ng/mL	255 (115, 395)	
Maximum inhibition (I_max_)	%	89.3 (87.3, 91.4)	
Hill coefficient		3.08 (0.555, 5.61)	
Residual variability	PU	23.8 (16.1, 31.5)	3.6
	%CV	21.9 (17.2, 26.6)	3.6
BSV in baseline dermal blood flow	%CV	28.3 (23.3, 33.2)	5.4
BSV in IC_50_	%CV	94.1 (68.1, 120)	12.2
Residual error	%CV	38.7 (27.1, 50.2)	33.3

95% CI = 95% confidence interval as estimated by the Wald’s test of mean estimate ±1.96·standard error

*BSV* Between-subject variability expressed as %CV, *CV* Approximates coefficient of variation, *PU* Perfusion unit

### Model-Based Simulations

**Fig. 5:**
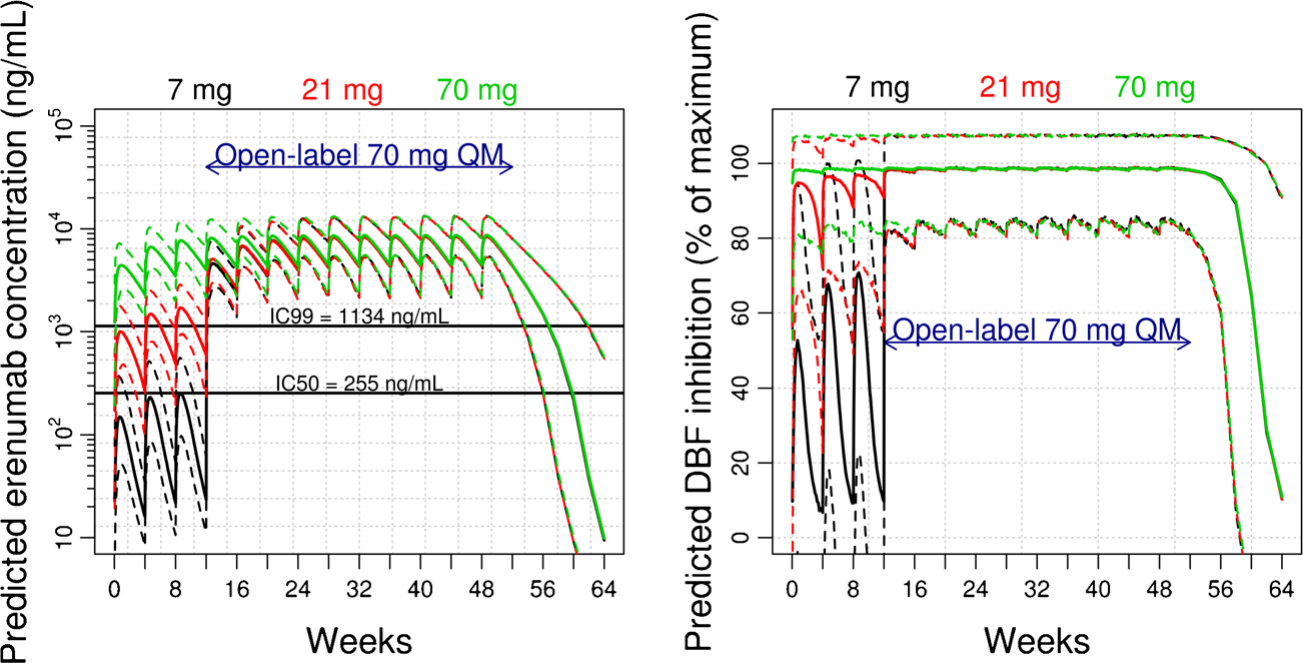
Predicted erenumab pharmacokinetics (PK) (*left panel*) and dermal blood flow (DBF) inhibition (*right panel*) for the selected doses in the phase 2 trial. Solid lines represent median population predictions; dashed lines represent 90% prediction intervals that account for total variability in PK or DBF response. The predicted DBF inhibition is expressed as percentage of the maximum possible inhibition (percent of I_max_ [89%]). QM, monthly.

Model-based simulation was performed to support dose selection for a migraine prevention study in subjects with episodic migraine. PK-PD simulations for dose selection was based on the assumptions that DBF inhibition was predictive of migraine efficacy (e.g., reduction of monthly migraine days) and maximum DBF inhibition was required for efficacy. Figure 5 shows the simulation results for 3 selected dose levels of erenumab at monthly dosing intervals. Based on the PK-DBF relationship, 21 mg SC every 4 weeks (Q4W) is expected to place more than 50% of subjects with concentrations above IC_99_ (e.g., concentration at 99% of maximum DBF inhibition) after the second dose. This regimen is projected to be the minimum migraine efficacious dose. At 70 mg SC Q4W, all subjects are expected to be above the mean IC_99_ within the first dose and this dose regimen is projected to have higher migraine efficacy than 21 mg. Lastly, 7 mg SC Q4W would provide the migraine efficacy information below the mean IC_50_, where 50% of DBF inhibition is achieved. The percentage of DBF inhibition relative to maximum inhibition provides further information about variability and duration of response (Fig. 5, right panel). At 70 mg SC Q4W, 100% of the maximum DBF inhibition had already been achieved for the entire duration of the study. Therefore, migraine efficacy (or reduction of migraine days) was expected to be maintained during the dosing interval.

## Discussion

We have described the PK of erenumab in healthy subjects and migraine patients using a mechanism-based TMDD model under the Qss assumption. The model successfully explained the dose/concentration-dependent elimination of erenumab, which was assumed to be mediated by elimination of the erenumab–receptor complex. We also characterized the PD effect of erenumab by linking the time course of erenumab serum concentrations to the time course of the inhibition of CIDBF.

According to model estimations, the mean absorption time was approximately 2 days and the estimated absorption half-life was 1.6 days, suggesting SC absorption was complete in approximately 8 days. The estimate of SC bioavailability for erenumab is 74% (95% CI: 66–85%), which is within the reported range for most mAb therapeutics (50–100%) [17]. Similar to other mAb therapeutics with nonlinear PK, erenumab exhibits TMDD behavior that is typically described by two parallel antibody elimination pathways: a slow non-specific elimination pathway through the hepatic reticuloendothelial system (i.e., linear clearance), and a rapid saturable elimination pathway (i.e., nonlinear clearance) mediated by degradation or internalization of the erenumab-receptor complex. The population estimate of linear clearance is independent of erenumab concentrations and stays approximately constant at 0.214 L/day (95% CI: 0.191–0.243), which is very similar to the typical clearance of endogenous IgG (~0.21 L/day) [18], with a low BSV (21% CV). In contrast, nonlinear clearance is dependent on the target receptor density and the amount of erenumab bound to the receptors. As shown in Supplemental Fig. S2, total clearance of erenumab decreased significantly and approached linear clearance as erenumab concentrations increased. Consequently, it is expected that dose regimens ≥70 mg SC Q4W would produce concentrations that saturate the nonlinear elimination pathway at steady state (Supplementary Fig. S2).

Body weight is a common covariate found to explain variability in the PK parameters of mAbs [18]. In the current dataset with a body weight range of 49–104 kg, for every 10 kg increase in body weight, linear clearance and central volume of distribution increased by 11% and 14%, respectively. Other covariates such as sex and age were highly correlated with body weight and had no further contribution to the variability in PK parameters. The majority of migraine patients were female (21 of 28 with PK) and their PK parameters were not significantly different from those of healthy subjects.

Erenumab effectively inhibited CIDBF increase in both healthy subjects and migraine patients at doses of 7 mg or greater (Fig. 3). The relationship between erenumab serum concentrations and DBF inhibition was sigmoidal and reached a plateau at approximately 1134 ng/mL (Fig. 4). The erenumab concentration-DBF relationship is robust and maximum inhibition is sustained with repeated dosing, with no evidence of attenuation. An indirect response model did not significantly describe data better than a direct response model, suggesting that erenumab’s effect was immediate and there was no evidence of delay in DBF inhibition. Erenumab’s concentration effect on DBF inhibition was not significantly different between healthy subjects and migraine patients, indicating that the biomarker model (i.e., CIDBF increase) is translatable from healthy subjects to migraine patients. Although body weight had a significant effect on erenumab serum concentrations, at the clinical doses of interest (i.e., 21 mg and 70 mg SC Q4W), body weight did not have an impact on DBF inhibition since the concentrations were well above the required concentration for maximum DBF inhibition (i.e., IC_99_, Supplementary Fig. S8).

Erenumab is highly potent in inhibiting capsaicin-induced increases in DBF in humans, with an IC_50_ of 255 ng/mL or 1.7 nM estimated with excellent precision (95% CI: 115–395 ng/mL). Thus, compared with telcagepant, an oral CGRP receptor antagonist with an IC_50_ of 101 nM [19], erenumab is much more potent. Erenumab has a similar potency to MK-3207 (IC_50_ = 1.59 nM), another oral CGRP receptor antagonist [20]. At a minimum, an erenumab dose of 7 mg SC easily provided serum concentrations to achieve target coverage. The maximum possible DBF inhibition estimated by modeling was 89% (95% CI: 87.3–91.4), which is comparable with the estimated maximum inhibition of 92% achieved with telcagepant [19]. These findings are consistent with the fact that, next to CGRP, other bioactive mediators are most likely involved in neurogenic inflammation induced by the activation of TRPV1 receptors in humans. Because previous studies excluded a substantial involvement of vasodilating prostaglandins, nitric oxide, and Substance P, the identities of additional mediators remain to be determined [21].

Compared with mAbs in development that target the CGRP ligand, such as LY2951742 [6], TEV-48125, and ALD-403 [22] erenumab requires a lower concentration to achieve and maintain maximum CIDBF inhibition; and the magnitude of maximum CIDBF inhibition is higher. For example, ALD-403 required approximately 308 nM (IC_99_ = 46,163 ng/mL) to reach a CIDBF inhibition plateau of only 61%.

It is important to note that although erenumab potently inhibits the capsaicin-induced increase in DBF, the basal DBF is not affected. Consequently, although CGRP is a potent endogenous vasodilator, it does not seem to be involved in maintaining basal peripheral vascular tone. Likewise, it has also been reported that the small molecule CGRP receptor antagonists olcegepant and telcagepant do not affect resting tissue perfusion. Taken together, these data suggest that CGRP receptor antagonists may be devoid of cardiovascular side-effects under resting conditions.

The major benefit of establishing the relationship between erenumab concentration and DBF inhibition is the ability to predict the erenumab exposures, magnitude, and duration of peripheral target engagement, and their theoretical relationship to efficacy in the target population. This is the cornerstone of model-based drug development that is used to guide dose selection in phase 2 dose ranging studies. We used the erenumab concentration-DBF model to select dose regimens for a dose-ranging study design under the assumption that maximum DBF inhibition is required for migraine efficacy (Fig. 5). Three doses, 7, 21, and 70 mg SC monthly were selected to provide dose-response information. Based on the simulation results, both 21 mg and 70 mg SC monthly regimens were predicted to be effective doses in the episodic migraine population, with the 70 mg SC monthly regimen predicted to have better efficacy. The 7 mg SC dose was added to demonstrate a non-effective dose. Since these predictions of migraine efficacy were theoretical we took a conservative approach by selecting 70 mg as the highest dose regimen, which exceeded IC_99_ by 7-fold after the first dose and 10-fold after the third dose. Results from the dose-ranging study in episodic migraine patients demonstrated a separation from placebo for only the 70 mg SC monthly dose (not for lower doses of 7 mg and 21 mg) [23]. These results indicate that the CIDBF pharmacology model, despite being a well validated translational biomarker to provide evidence of peripheral target engagement, does not necessarily predict exposures needed to obtain anti-migraine efficacy [24].

Interestingly, a comparable observation was reported for telcagepant [19] in that the effective therapeutic doses in acute migraine clinical trials resulted in mean plasma concentrations that were approximately 2- to 4-fold higher than the IC_90_ in the capsaicin model, suggesting that larger doses than those producing maximal peripheral CGRP receptor inhibition are necessary for anti-migraine efficacy. Given these observations, it has been suggested that therapeutic efficacy in the treatment of migraine, traditionally thought to be a central nervous system (CNS) disorder, might require higher doses than expected based on peripheral target engagement. Indeed, if penetration in the CNS is needed, peripheral target engagement might not be representative of concentrations needed to achieve central target engagement, as mAb concentrations in the CNS are only ~0.1% of their serum concentrations [25]. However, increasing evidence suggests that central penetration is not needed to obtain anti-migraine efficacy. Recent PET data with telcagepant convincingly showed that, at therapeutic doses, no meaningful occupancy of CGRP-receptors was observed in the CNS [26]. This observation supports the concept that, to relieve migraine, it is sufficient for CGRP-blocking therapeutics, including mAbs, to act peripherally. However, this does not exclude the possibility that compounds acting both peripherally and centrally might have added value.

There are some limitations in the current analysis. The TMDD model implied that the erenumab nonlinear elimination pathway is mediated by its binding to the CGRP receptor. However, the density/amount of CGRP receptors in humans is not known; therefore, the PK parameters related to target-mediated elimination are data driven. The covariate effects evaluated in this PK-PD analysis are applicable only to the observed range of baseline characteristics in the included studies. Therefore, covariates that are found not significant in this analysis should be re-evaluated in population analysis where a broader population of subjects are included.

## Conclusions

The PK characteristics of erenumab are typical of mAb therapeutics that exhibit TMDD. Furthermore, the erenumab concentration-DBF relationship indicates that erenumab is highly potent against peripheral CGRP receptors and that its pharmacological effect is sustained with repeated administration. Erenumab shows maximum target engagement after single and repeated dosing, and has a long serum effective half-life at doses ≥70 mg SC. The established PK-PD relationship of erenumab provides a useful tool to select appropriate dose regimens and study design for future clinical studies in migraine patients.

## Acknowledgments and Disclosures

This study was funded by Amgen Inc. The authors thank the staff at the Centre for Clinical Pharmacology at University Hospitals of Leuven, especially Jo Van Effen and Marissa Herbots, for assisting in the experiments and data collection. The authors also acknowledge Adimoolam Narayanan of Amgen Inc. for programming support and study summaries. Lisa Humphries of Amgen Inc. and Martha Mutomba (on behalf of Amgen Inc.) provided formatting, editing, and submission support.

TV, LY, LH, and GV are employees of Amgen and may have Amgen stock/stock options; LSW, PM, and JSC were employees of Amgen at the time these analyses were conducted. JdH reports research grants from Abide, Amgen, Galderma, Genentech, GlaxoSmithKline, Janssen Research & Development, Lilly Chorus, MSD, Novartis, Sanofi Pasteur, UCB, and Vertex and reports consultancy for Ablynx, Amgen, Eli Lilly, Genentech, and UCB. AVH has declared no conflicts of interest.

## Electronic supplementary material

ESM 1 (DOCX 2549 kb)

## Abbreviations

CGRP: Calcitonin gene-related peptide
CIDBF: Capsaicin-induced dermal blood flow
DBF: Dermal blood flow
IC_50_: Concentration providing 50% inhibitory effect
IC_99_: Concentration providing 99% inhibitory effect
IV: Intravenous
MAD: Multiple-ascending dose
PD: Pharmacodynamic
PK: Pharmacokinetic
SAD: Single-ascending dose
SC: Subcutaneous
TMDD: Target-mediated drug disposition
TRPV1: Transient receptor potential vanilloid type 1 receptor
VPC: Visual predictive check
