# Supplementary material for: Pharmacokinetic-Pharmacodynamic Relationship of Erenumab (AMG 334) and Capsaicin-Induced Dermal Blood Flow in Healthy and Migraine Subjects
Source: Pharm Res. 2017 Jun 7;34(9):1784–95. doi: 10.1007/s11095-017-2183-6 (PMC5533838; doi:10.1007/s11095-017-2183-6)
Supplement: Supplementary file 1 — (DOCX 2549 kb) [file 11095_2017_2183_MOESM1_ESM.docx]

# SUPPLEMENTARY INFORMATION

**Pharmacokinetic-Pharmacodynamic Relationship of Erenumab (AMG 334) and Capsaicin-Induced Dermal Blood Flow in Healthy and Migraine Subjects**

**Journal:** *Pharmaceutical Research*

**Authors:** Thuy Vu^1^ • Peiming Ma^2^ • Jiyun Sunny Chen^3^ • Jan de Hoon^4^ • Anne Van Hecken^4^ • Lucy Yan^1^ • Liviawati Sutjandra Wu ^1^• Lisa Hamilton^5^ • Gabriel Vargas^1^

**Affiliations:** ^1^Clinical Pharmacology, Modeling and Simulation, Amgen Inc., Thousand Oaks, California, USA; ^2^Clinical Pharmacology, GSK R&D, Shanghai, China; ^3^Medivation, San Francisco, California, USA; ^4^Center for Clinical Pharmacology, University Hospitals of Leuven, Leuven, Belgium; ^5^Global Biostatistical Sciences, Amgen Limited, Uxbridge, England, UK

**Corresponding Author:**

Thuy Vu, PharmD

Amgen Inc.

One Amgen Center Drive

Thousand Oaks, California 91320-1799

USA

Phone: (805) 447-4276

Fax: (805) 375-6165

E-mail: thuy@amgen.com

# SUPPLEMENTARY METHODS

## PK and PD Model Development

Statistical Model

Random effects η as between-subject variability (BSV) in parameters were considered and assumed to be log-normally distributed with covariance matrix Ω. The PK model was fitted to serum concentrations of erenumab (AMG 334) after natural-logarithmic transformation: ln(Y_obs,ij_) = ln(Y_pred,ij_) + ε, where Y_obs,ij_ and Y_pred,ij_ are observed and predicted concentrations, respectively, of a subject i at time j, and ε is residual unexplained variability normally distributed with mean 0 and variance σ^2^. The PD model was fitted to untransformed ΔDBF data: Y_obs,ij_ = Y_pred,ij_ + W⋅ε, where W was estimated as standard deviation (additive error), %CV (proportional error), or a combination, and ε was fixed to follow a standard normal distribution.

Covariate Analyses

After a base model was determined, body weight, age, sex, and type of population (healthy subjects vs migraine patients) were tested as possible sources of variability on clearance and volume of distribution parameters. Similarly, age and population type were tested on PD parameters (e.g., baseline DBF, I_max_, and IC_50_). Covariates were evaluated in a stepwise manner with forward addition and backward elimination to remove potential confounding factors. Covariate models were evaluated for statistical significance according to model selection criteria. Continuous covariates were evaluated using power equations after centering at the median:

$$P_{ij}=P_{j}\cdot\left(\frac{X_{i}}{Median(X_{i})}\right)^{\beta_{CONT}}$$

where β_CONT_ related the continuous covariate to the median or typical population value P_j_; X_i_ was the covariate value for the i^th^ individual, and median(X_i_) was the median of the covariate X_i_ or generally accepted typical value (e.g., 70 kg for body weight). Dichotomous covariates were entered into the model as an index variable and the fractional change relative to the reference group was estimated:

$$P_{ij}=P_{j}\cdot e^{\left(\beta_{CAT}\cdot X_{ij}\right)}$$

thus, when X_ij_ = 1, P_ij_ = P_j_ e^(βCAT)^ and when X_ij_ = 0, P_ij_ = P_j_. Missing values for the continuous covariates were imputed using the median value in each dataset, and missing values for categorical covariates were analyzed as an independent category.

Model Evaluation Criteria

The improvement in the fit obtained for each model was assessed in several ways. First, NONMEM-generated minimum values of the objective function (MVOF) were used to perform the likelihood ratio test. For nested models, a decrease in MVOF of 10.83 (to reach a statistical significance level of 0.001) was set for including a fixed effect. In addition, the improvement in the fit was assessed by the reduction in the BSV and residual variability, the precision in parameter estimates, and the examination of diagnostic plots and shrinkage (1). Akaike Information Criterion (AIC) was used when competing models were non-nested (e.g., one- vs two-compartment model). A model with minimum AIC was selected among competing models (2).

Parameter imprecision was reported as 95% confidence interval around mean parameter estimates based on model standard errors or bootstrap estimation. Internal model evaluation of model structure was performed using visual predictive check and standard diagnostic plots (i.e., population predictions vs observed, individual predictions vs observed, and residuals).

Simulations

Using the best fitted PK-PD model, simulations for a phase 2 dose-ranging study were conducted to explore erenumab dose regimens that would potentially provide a range of clinical efficacy (i.e., minimal, 50%, and 100% reductions in migraine days) in migraine patients based on the degree of DBF inhibition. The study design included Q4W dosing for a duration of 12 weeks, followed by open-label Q4W dosing for an additional 40 weeks. Covariates from the phase 1 population were randomly sampled and assigned to each dosing cohort (100 subjects per cohort). To generate overall variability in the predictions, 100 replications of the simulation were generated. Effect of uncertainty in parameters on trial simulation was assumed to be minimal compared to total variability in parameters and was therefore ignored. Predicted mean PK and DBF inhibition profiles along with 90% prediction intervals were presented for phase 2 dose selection.

To assess the effect of body weight on the time course of CIDBF, deterministic simulations were performed for 3 monthly (Q4W) doses of erenumab using the 25^th^, 50^th^ and 75^th^ percentiles of the observed body weights. Erenumab concentrations and percent of DBF inhibition time profiles were plotted for illustration.

Software

A nonlinear mixed-effects modeling method was implemented by maximizing the log-likelihood using the approach of first-order conditional estimation with interaction with the NONMEM software Version 7.2.0 (ICON Development Solutions; Ellicott City, MD, USA). Graphical and all other statistical analyses, including the evaluation of NONMEM outputs, were performed using R program version 3.0.2 (3).

# SUPPLEMENTARY REFERENCES

1. Karlsson, MO & Savic, RM. Diagnosing model diagnostics. Clin Pharmacol Ther. 2007;82:17–20.
2. Akaike, H. A new look at the statistical model identification. IEEE Trans Automat Contr. 1974;19:716–723.
3. The R Foundation for Statistical Computing. R: A Language and Environment for Statistical Computing. Version 3.0.2 (The R Foundation for Statistical Computing, Vienna, 2013). Available from: <https://www.r-project.org/>. [Website].

# SUPPLEMENTARY FIGURES

**Supplementary Fig. S1** Pharmacokinetic model schematic. IV, intravenous; SC, subcutaneous; F = bioavailability; k_a_, k_cp_, and k_pc_, are absorption rate, transfer rate from central to peripheral, and transfer rate from peripheral to central, respectively; A_c_, A_p_ – unbound erenumab amounts in central and peripheral compartments, respectively; V_c_, V_p_ – volumes of distribution in central and peripheral compartments, respectively; R – target receptor amount in central compartment; RA – bound erenumab – receptor complex amount; k_syn_, k_deg_, k_int_, and K_ss_ are synthesis rate, degradation rate, internalization rate and quasi-steady-state binding constant, respectively.

Supplementary Fig. S2 Relationship between unbound erenumab concentrations and clearances.


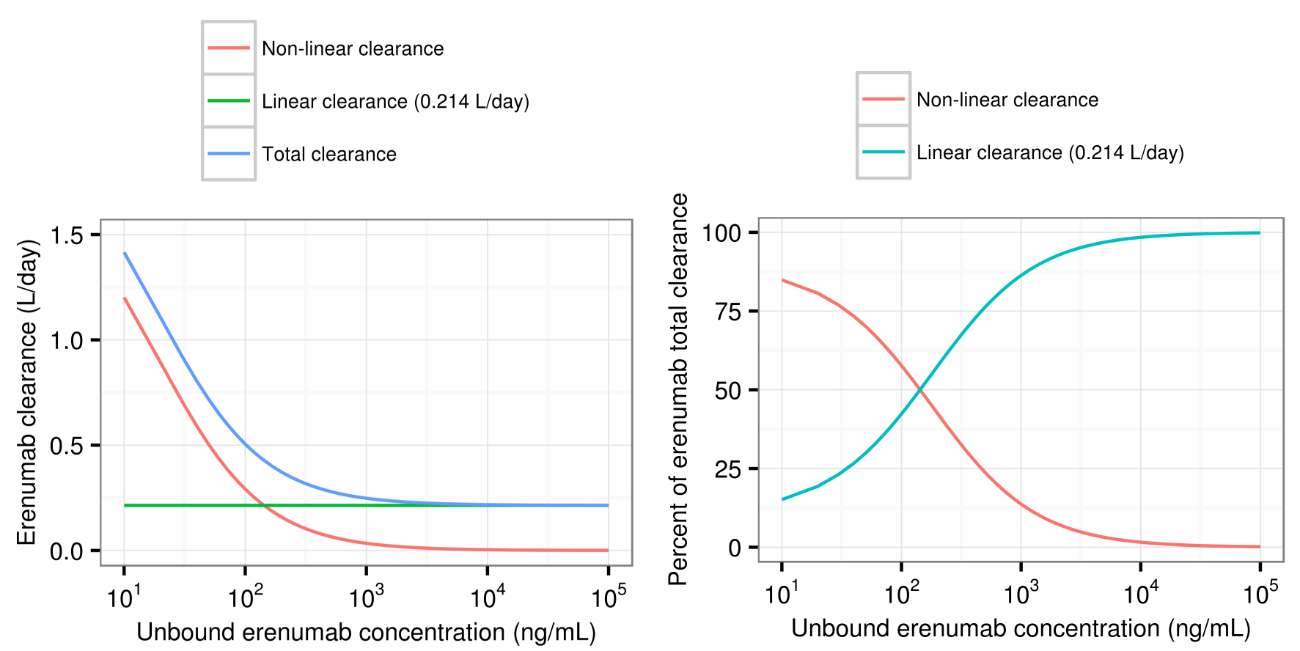


Supplementary Fig. S3 Similar time courses of erenumab serum concentrations and dermal blood flow (DBF) inhibition were observed between healthy subjects and migraine patients. Data are shown as observed mean ± standard deviation. Q4W, every 4 weeks; SC, subcutaneous.

**
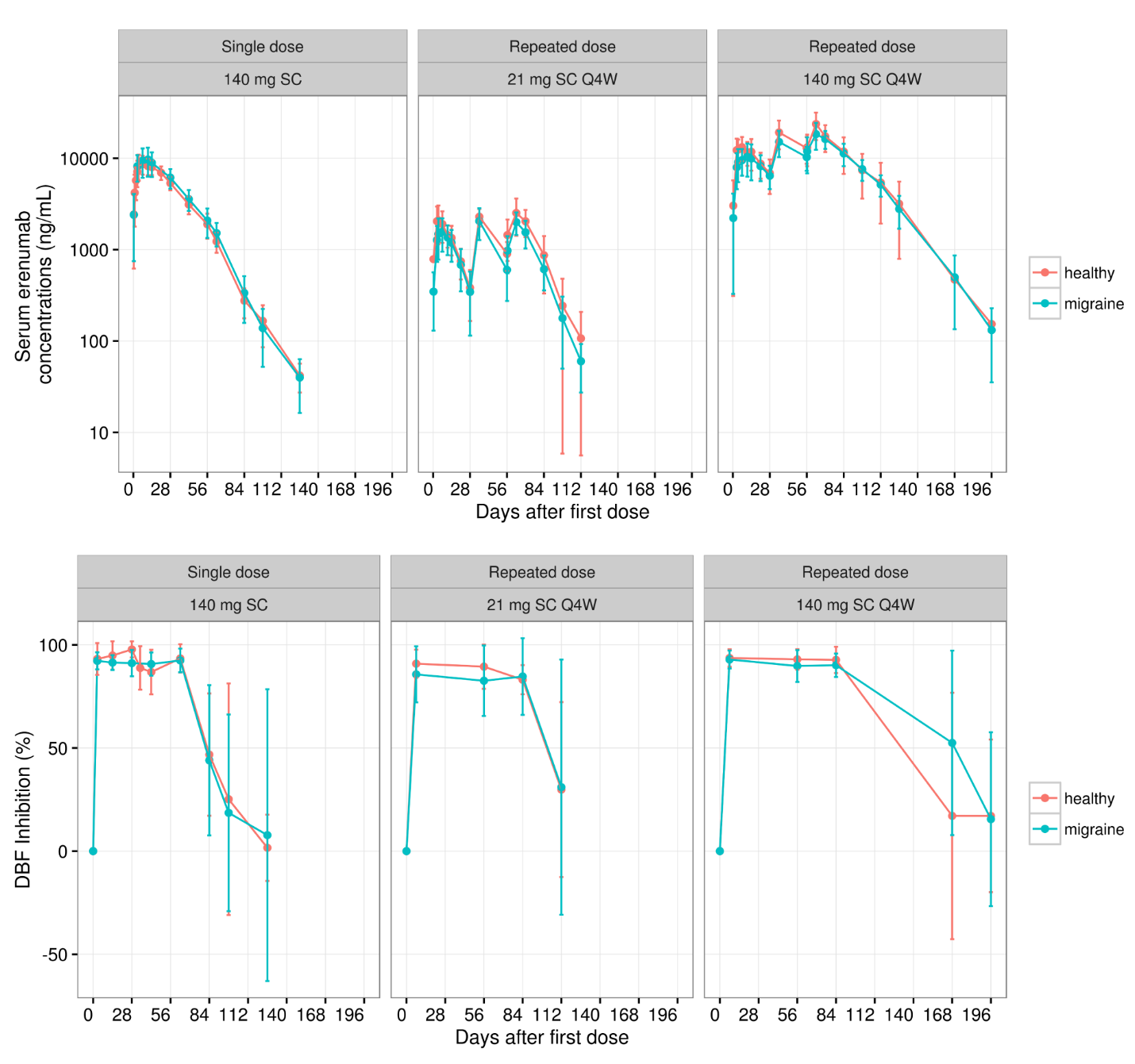
**

Supplementary Fig. S4 General goodness-of-fit for the final pharmacokinetic model for erenumab serum concentrations. IV, intravenous; Q4W, every 4 weeks; SC, subcutaneous.


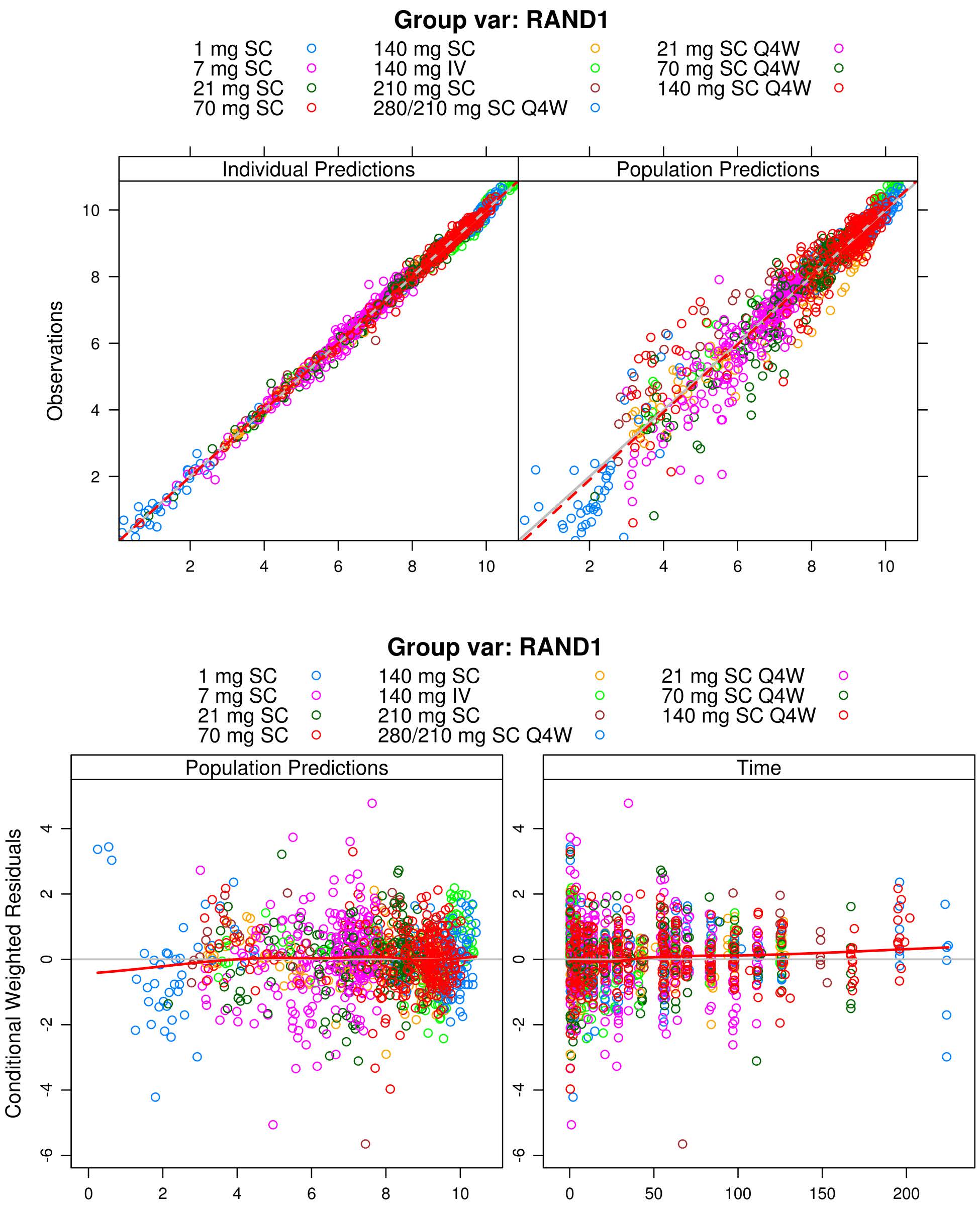


Supplementary Fig. S5 General goodness-of-fit for the final pharmacodynamic model for dermal blood flow measurements for erenumab. IV, intravenous; Q4W, every 4 weeks; SC, subcutaneous.


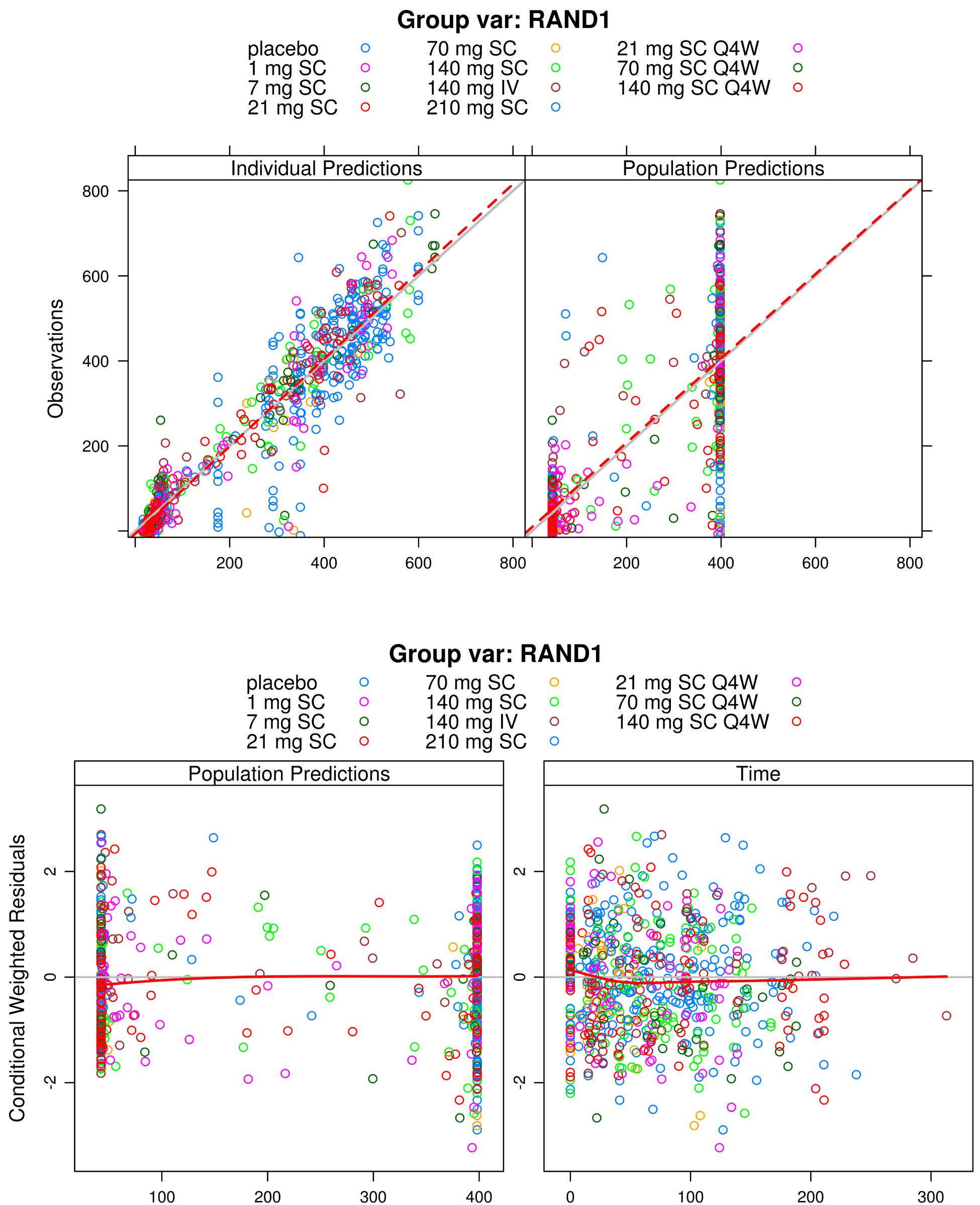


**Supplementary Fig. S6** Prediction-corrected VPC for erenumab (a) pharmacokinetics and (b) dermal blood flow models (red lines are 5^th^, 50^th^ and 95^th^ percentiles of observed data; blue lines are 5^th^, 50^th^ and 95^th^ percentiles of model predictions with the corresponding 95% confidence intervals). VPC, visual predictive check.


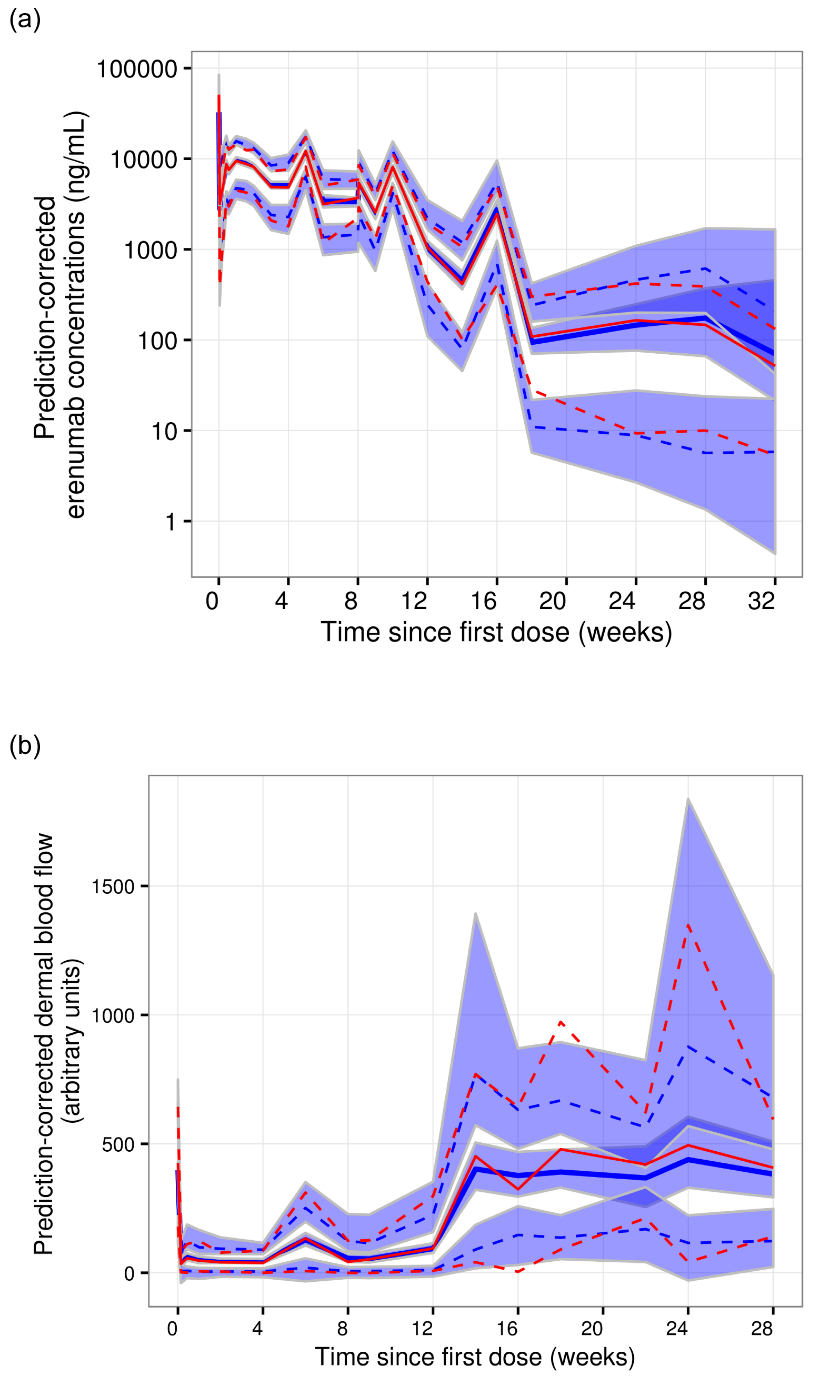


Supplementary Fig. S7 Observed repeated measurements of dermal blood flow (DBF) before and after capsaicin (CAP) challenge in the erenumab highest single-dose (210 mg SC) and highest multiple-dose (140 mg SC Q4W) cohorts. Data are shown as observed mean+standard deviation. Q4W, every 4 weeks; SC, subcutaneous.

**
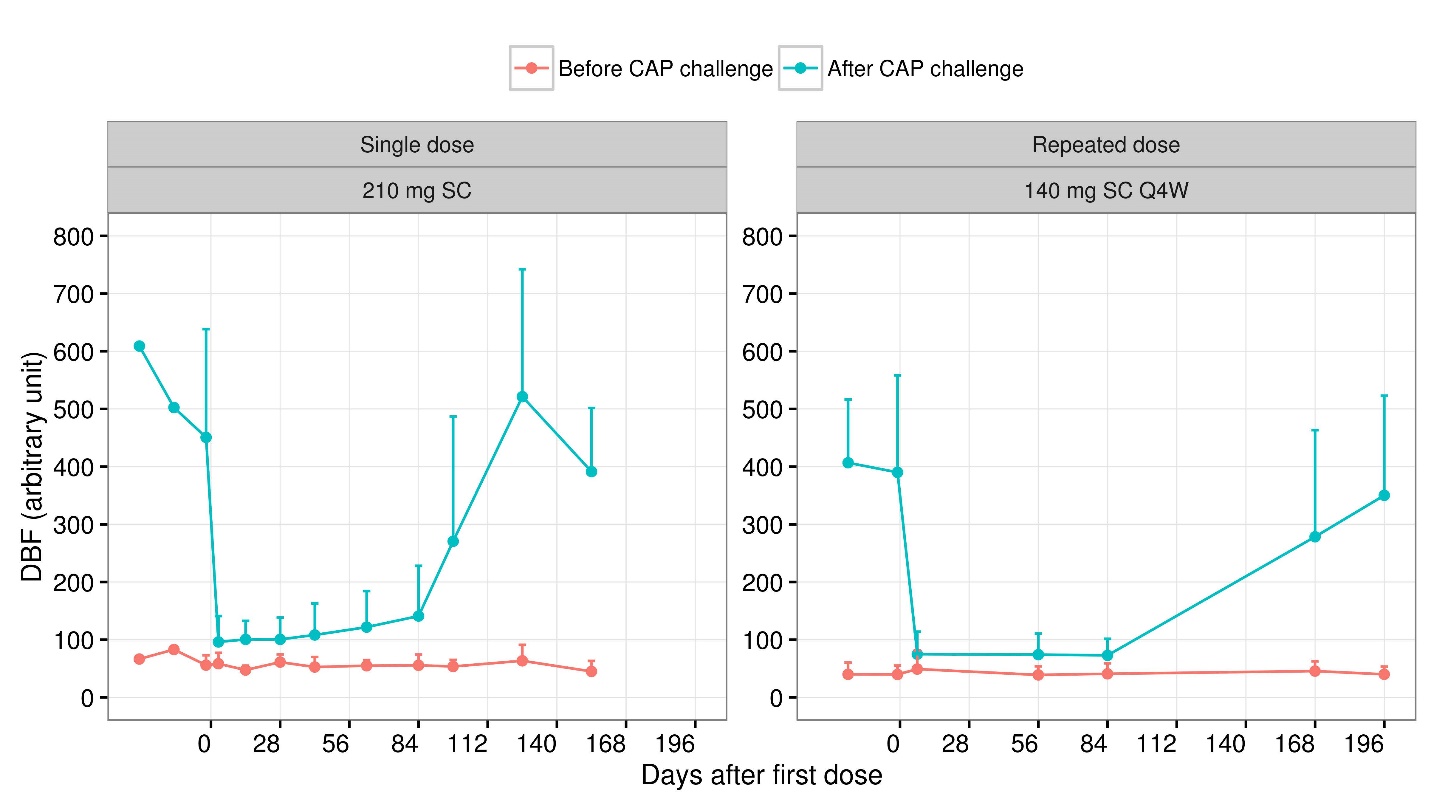
**

**Supplementary Fig. S8** Illustration of the indirect effect of body weight on dermal blood flow inhibition through the effect of body weight on erenumab pharmacokinetics for 3 monthly erenumab doses. Simulations were based on the 25^th^, 50^th^, and 75^th^ percentiles of body weights (i.e., 68, 75, and 84 kg, respectively) observed in phase 1 studies. Q4W, every 4 weeks; SC, subcutaneous.

**
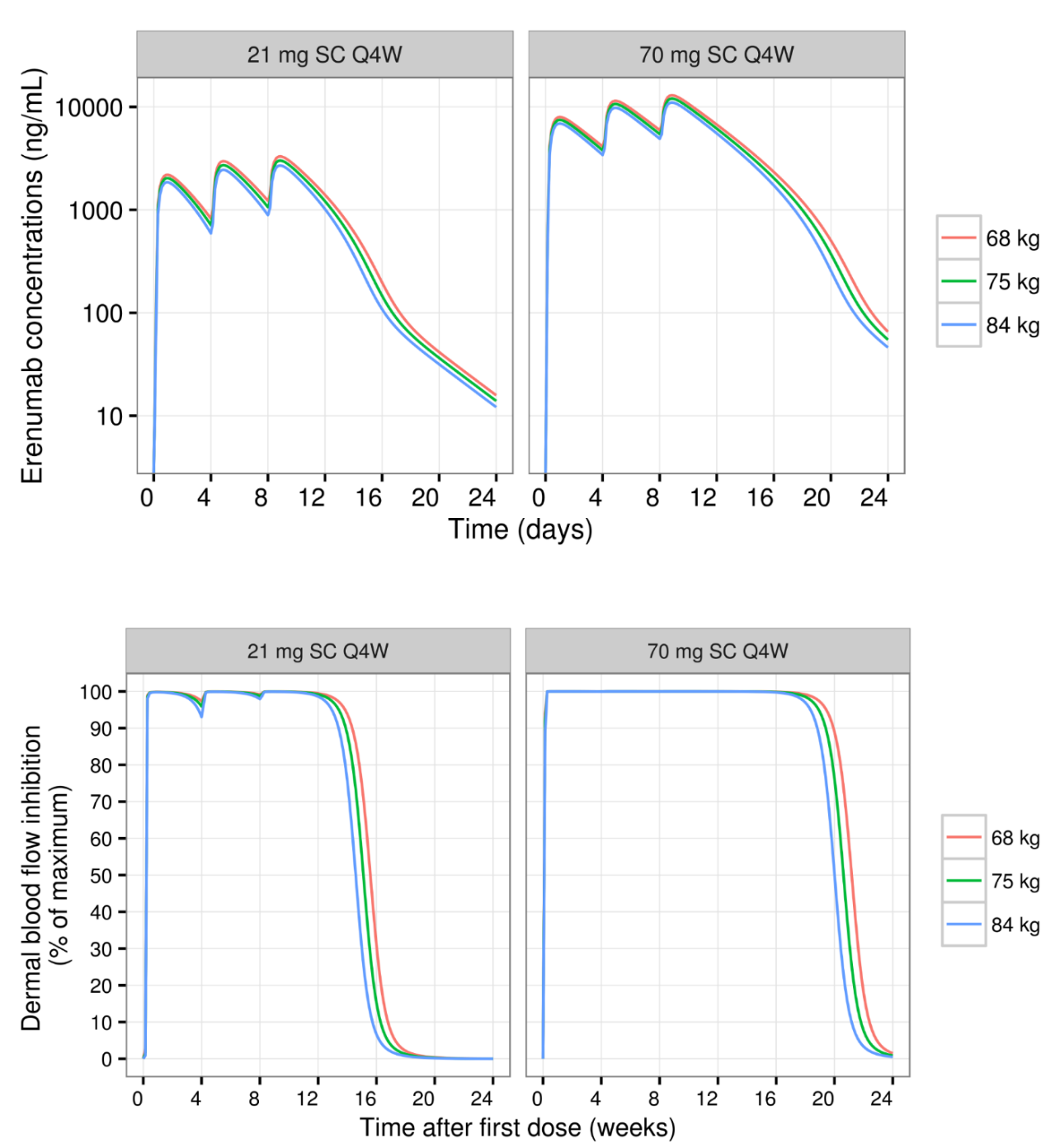
**
